# Chloroquine Stimulates Cl^−^ Secretion by Ca^2+^ Activated Cl^−^ Channels in Rat Ileum

**DOI:** 10.1371/journal.pone.0087627

**Published:** 2014-01-30

**Authors:** Ning Yang, Zhen Lei, Xiaoyu Li, Junhan Zhao, Tianjian Liu, Nannan Ning, Ailin Xiao, Linlin Xu, Jingxin Li

**Affiliations:** 1 Department of Physiology, Shandong University School of Medicine, Jinan, China; 2 Department of Anesthesiology, Qilu Hospital, Shandong University, Jinan, China; Bascom Palmer Eye Institute, University of Miami School of Medicine, United States of America

## Abstract

Chloroquine (CQ), a bitter tasting drug widely used in treatment of malaria, is associated gastrointestinal side effects including nausea or diarrhea. In the present study, we investigated the effect of CQ on electrolyte transport in rat ileum using the Ussing chamber technique. The results showed that CQ evoked an increase in short circuit current (*I_SC_*) in rat ileum at lower concentration (≤5×10^−4 ^M ) but induced a decrease at higher concentrations (≥10^−3^ M). These responses were not affected by tetrodotoxin (TTX). Other bitter compounds, such as denatoniumbenzoate and quinine, exhibited similar effects. CQ-evoked increase in *I_SC_* was partly reduced by amiloride(10^−4^ M), a blocker of epithelial Na^+^ channels. Furosemide (10^−4^ M), an inhibitor of Na^+^-K^+^ -2Cl^−^ co-transporter, also inhibited the increased *I_SC_* response to CQ, whereas another Cl^−^ channel inhibitor, CFTR(inh)-172(10^−5^M), had no effect. Intriguingly, CQ-evoked increases were almost completely abolished by niflumic acid (10^−4^M), a relatively specific Ca^2+^-activated Cl^−^ channel (CaCC) inhibitor. Furthermore, other CaCC inhibitors, such as DIDS and NPPB, also exhibited similar effects. CQ-induced increases in *I_SC_* were also abolished by thapsigargin(10^−6^M), a Ca^2+^ pump inhibitor and in the absence of either Cl^−^ or Ca^2+^ from bathing solutions. Further studies demonstrated that T2R and CaCC-TMEM16A were colocalized in small intestinal epithelial cells and the T2R agonist CQ evoked an increase of intracelluar Ca^2+^ in small intestinal epithelial cells. Taken together, these results demonstrate that CQ induces Cl*^−^* secretion in rat ileum through CaCC at low concentrations, suggesting a novel explanation for CQ-associated gastrointestinal side-effects during the treatment of malaria.

## Introduction

Chloroquine (CQ) is a drug commonly used for prevention and treatment of malaria. Use of this drug has been expanded for the treatment of other diseases, such as rheumatoid arthritis, systemic lupus erythematous and other related disorders. CQ is usually well tolerated,however, gastrointestinal side effects including nausea or diarrhea have been described [1], [2], [3], [4]. The underlying mechanisms for these side effects are unclear.

CQ is a synthetic bitter-tasting compound. Many bitter-taste receptors,which are believed to function as gatekeepers in the oral cavity to detect and prevent the ingestion of poisonous bitter-tasting compounds, are expressed in mammalian testis [5] and lung [6]. In addition, bitter taste receptors are expressed in the intestinal tract,which is involved in sensing of food components [7], [8], [9], [10], [11], [12], [13]. Kaji et al reported that the bitter compound, 6-PTU, evoked anion secretion in the large intestines of humans and rats [8]. Intestinal transepithelial ion transport is regulated by diverse systems, including the enteric nervous system (ENS) and a variety of gut hormones and cytokines, responding to mechanical and chemical stimuli [14]. In this study, we investigated the effect of CQ on electrolyte transport in rat ileum as assessed with the ussing chamber technique. Our results showed that CQ induces Cl*^−^* secretion in rat ileum through CaCC at low concentraions and that these effects might not involve the neural pathway. These findings provide a novel explanation for the gastrointestinal side-effects of CQ-associated with the treatment of malaria.

## Materials and Methods

### Animals and Tissue Preparation

All experimental procedures were conducted in accordance with the Guidelines for the Care and Use of Laboratory Animals of Shandong University, and the study was approved by the Medical Ethics Committee for Experimental Animals, Shandong University, China (number ECAESDUSM 2012029). Adult male Wistar rats (Animal Center of Shandong University, China), weighing between 200 and 250g, were used for this study. Animals were fasted overnight,but permitted free access to water before experiments. They were anesthetized with ether and decapitated. Tissue preparation was according to that described previously [15]. Segments of ileum were cut along the mesenteric border, and luminal contents were gently removed. Tissues were pinned flat on a Sylgard-lined Petri dish with mucosal surface facing down. To obtain mucosal-submucosal preparations, serosa and muscularis were gently stripped away. During preparation, tissues were bathed in ice-cold Krebs solution(bathing solution) and continuously oxygenated with a gas mixture of 95%O_2_ and 5%CO_2_. The Krebs solution contained (in mM): 120.6 NaCl, 5.9 KCl, 2.5 CaCl_2_,1.2 KH_2_PO_4_, 1.2 MgCl_2_, 15.4 NaHCO_3_ and 11.5 glucose.

### Short-circuit Current Measurement

Short-circuit current (*I_SC_*) was measured *in vitro* in Ussing chambers. The tissue preparations were mounted between the 2 halves of the Ussing chambers (exposed area of 0.50 cm^2^), equipped with water-jacketed gas lifts. They were bathed on both sides with 5 mL Krebs solution, gassed with 95% O_2_ and 5% CO_2_, pH adjusted to 7.4, and maintained at 37°C by circulating the solution through a reservoir during the experiments. The tissue was continuously voltage-clamped to zero potential difference by the application of external current, with compensation for fluid resistance. The baseline value of the electrical parameters was determined as the mean over the 3 min immediately prior to drug administration. The tissues were allowed to equilibrate to these conditions for approximately 30 min to stabilize the *I_SC_* prior to the addition of drugs. The transepithelial potential difference for each preparation was measured with Ag/AgCl reference electrodes (P2020S; Physiologic Instruments, San Diego, Calif) connected to a preamplifier that was, in turn, connected to a voltage clamp amplifier (VCC MC4; Physiologic Instruments, San Diego, Calif). The change in the short circuit current (Δ*I_SC_*) was calculated on the basis of the value before and after the stimulation and was normalized as the current per unit area of epithelium (µA/cm2). To check tissue viability, tissues were stimulated by carbachol(CCh).

The bitter compounds, CQ, denatoniumbenzoate, and quinine were added to the serosal bathing solution and changes in *I_SC_* were measured. With the exception of quinine, 5-nitro-2-(3-phenylpropylamino) benzoic acid(NPPB) and 4,4′-diisothiocyanatostilbene-2,2′-disulphonic acid(DIDS) (dissolved inDMSO), each drug was dissolved in distilled water and added to the bath to provide the desired molarconcentration. Amiloride(10^−4^M), CFTR(inh)-172(10^−5^M), niflumic acid(10^−4^M), furosemide (10^−4^M), thapsigargin(10^−6^M), Cl^–^ free solution, and Ca^2+^ free solution were used to investigate the ion component of CQ-evoked *I_SC_* changes. The Cl^–^ free solution contained (in mM): 117 Na-gluconate, 4.7 K-gluconate, 8 Ca-(gluconate)_2_,1.2 Mg-(gluconate)_2_,1.2 NaH_2_PO_4_, 25 NaHCO_3_, and11 glucose. The Ca^2+^-free EDTA solution contained (in mM): 117 NaCl, 4.7 KCl, 2.5 MgCl_2_.6H_2_O,1.2 NaH_2_PO_4_, 25 NaHCO_3_, 3 EDTA and11 glucose. These solutions were bubbled with a gas mixture of 95% O_2_ and 5% CO_2_ and buffered at pH7.2–7.4. Tetrodotoxin (TTX-10^−6^M) was used to test the influence of neural pathway.

### Immunohistochemistry

IEC-18 cells were used after 48h in culture. Specifically,the cells were fixed in 4% paraformaldehyde for 10 min and rinsed three times with th PBS,and were blocked by 10% donkey serum for 1h at room temperature. Then, the cells were incubated with mouse anti-TMEM16A(1∶100; Santa Cruz) or anti-TAS2R10(1∶300;Abcam) diluted with 10% donkey serum over night at 4°C. Next, the cells were incubated with. Alexa Fluor 568-conjugated donkey anti-rabbit(1∶5000; Invitrogen) and Alexa Fluor 488-conjugated donkey anti-mouse IgG (1∶5000;Invitrogen) at room temperature for 1h followed by washes with PBS. The DAPI (1∶1,000) was used to stain the nucleus. The coverslips were mounted with75% glycerol.Negative controls were stained without primary antibodies.

### Chemical

All drugs were purchased from Sigma-Aldrich Corp.(St.Louis,MO,USA). With the exception of CFTR(inh)-172 and thapsigargin (dissolved in DMSO), each drug was dissolvedin distilled water. The volume of dissolved drugs in H_2_O or DMSO added to the bathing solutions did not exceed 15 µl (0.3% of bathing solution). The 0.3% DMSO did not affect *I_SC_* in rat tissues that was used in our study.

### Data Analysis and Statistics

All data are expressed as means ±SE.The n values represent the numbers of animals.

One-way ANOVA or unpaired Student’s *t*-tests were used to determine whether there were significant differences in basal electrical parameters among the tissue elements. *P*<0.05 was considered statistically significant.

## Results

### CQ Evoked an Increase in *I_SC_* in Rat Ileum

**Figure 1:**
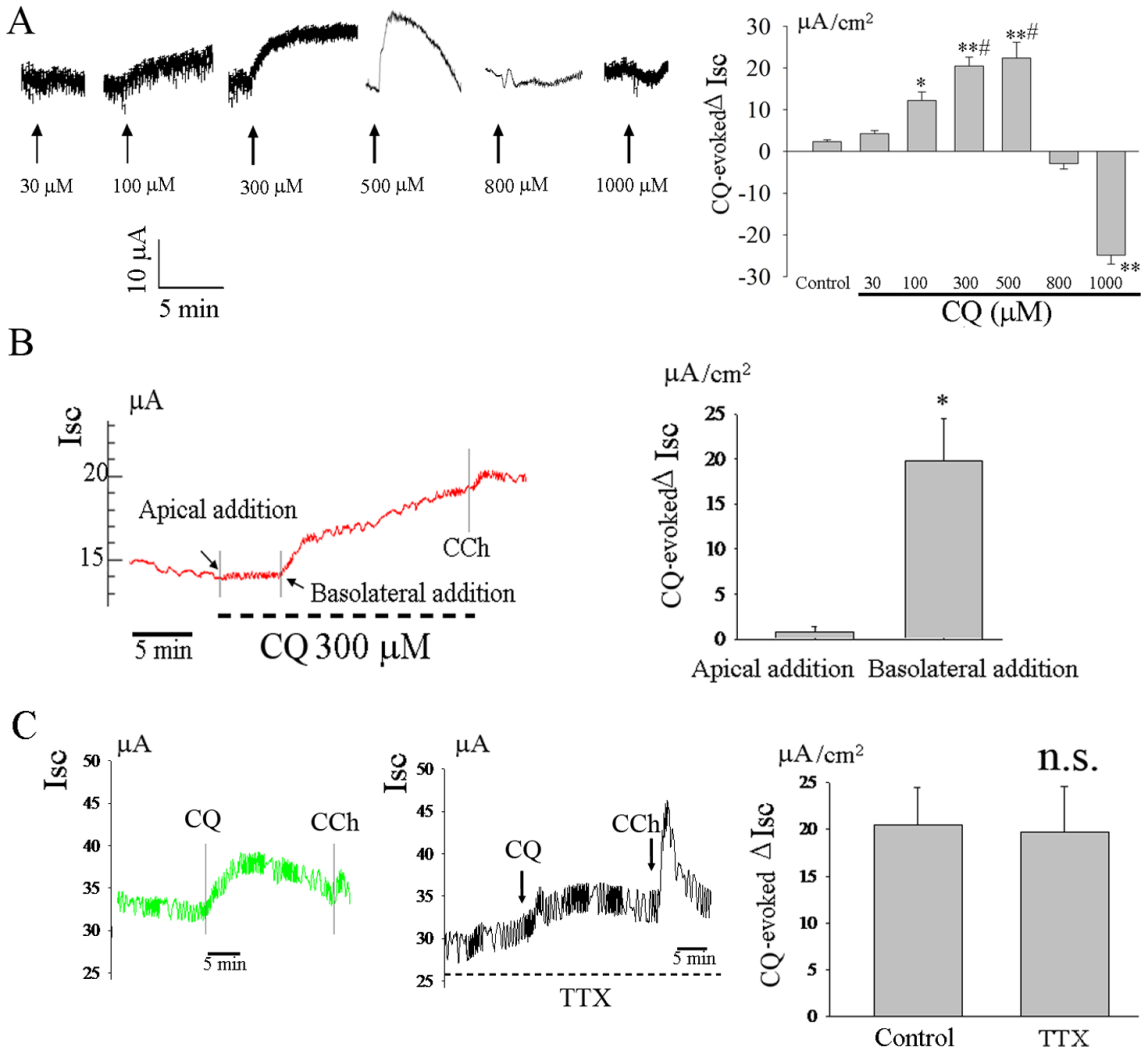
CQ evoked an increase in *I_SC_* in rat ileum. CQ dose-dependently increased basal *I_SC_* at low concentrations(≤5×10^−4 ^M), however, it markedly decreased basal *I_SC_* at high concentrations (≥10^−3^ M) (n = 6, A). The serosal addition of CQ (3×10^−4^M) increased basal *I_SC_*, whereas CQ to mucosal bathing solution had no effect on basal *I_SC_* (n = 6,B)_._ The CQ-induced increase in *I_SC_* was not influenced by TTX(n = 4,C). *P<0.05; **P<0.01 compared with control or apical addition; ^#^ *P*<0.05 compared with 100 µM group; n.s: no significance by the one-way ANOVA or unpaired *t*-test.

CQ dose-dependently increased basal *I_SC_* at low concentrations(≤5×10^−4 ^M), however, it markedly decreased basal *I_SC_* at high concentrations (≥10^−3^ M) (n = 6, Figure 1A). To further investigate the underlying mechanism of this CQ-induced *I_SC_* response in rat ileum, CQ (3×10^−4 ^M) was added to either the mucosal or serosal bathing solutions. The CQ-induced *I_SC_* response was completely absent when CQ was added to the mucosal bathing solution (n = 6, Figure 1B)_._

### Effect of TTX on the CQ-evoked Increases *I_SC_* in Rat Ileum

The ENS plays an important role in the regulation of intestinal epithelial ion transport. To investigate the involvement of the ENS in the serosal CQ-induced *I_SC_* response. TTX(10^−6^M) was added to the serosal bathing solution 15 min before the addition of CQ. TTX did not affect the CQ-induced increase in *I_SC_* (n = 4, Figure 1C).

### Effects of Amiloride, CFTR(inh)-172, Furosemide, Niflumic Acid, DIDS and NPPB on CQ -evoked Increase in *I_SC_* in Rat Ileum

**Figure 2:**
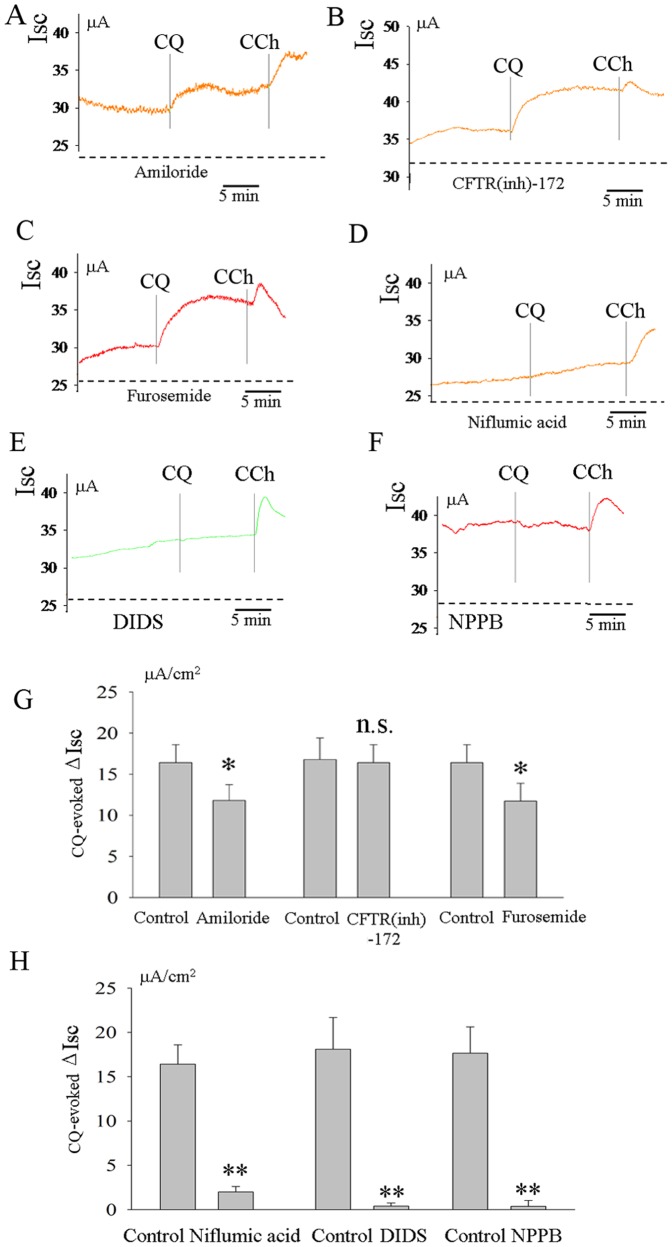
Effects of amiloride, CFTR(inh)-172, niflumic acid and furosemide on CQ-evoked increase in I_SC_ in rat ileum. The CQ-evoked increase in *I_SC_* was partly inhibited by a blocker of epithelial Na^+^ channels, amiloride(10^−4^M)(n = 7, A and G). Cl^−^ channel inhibitor CFTR(inh)-172(10^−5^M) did not affect this CQ-evoked response (n = 5,B and G)_._ An inhibitor of Na^+^-K^+^ -2Cl^−^ cotransporter, furosemide (10^−4^M) also reduced the CQ-evoked increase in *I_SC_* (n = 4, C and G). Niflumic acid (10^−4^M), DIDS and NPPB almost completely abolished the CQ-evoked increase in Isc(n = 9, D-F and H). *P<0.05; **P<0.01;n.s: no significance by unpaired *t*-test.

These experiments were designed to investigate the ion components of *I_SC_* induced by CQ.Two preparations of ileum from one rat were used for the control and experimental groups. Amiloride (10^−4 ^M), a blocker of epithelial Na^+^ channels, was added to mucosal bathing solution 15 min before the serosal application of CQ and partly reduced the CQ-evoked *I_SC_* from 16.5±2.3 µA/cm^2^ in the control group to 12.3±2.1 µA/cm^2^ in the experimental group (*P*<0.05 by paired t-test, n = 7, Figures 2A and 2G). CFTR(inh)-172, an inhibitor of cystic fibrosis transmembrane conductance regulator (CFTR), was also added to the mucosal bathing solution 15 min before the application of CQ, but did not affect the CQ-evoked increase in *I_SC_* (n = 5, Figures 2B and 2G)_._ The serosal addition of the Cl^−^ transporter inhibitor, furosemide (10^−4^M), partly reduced the CQ-evoked increase (from 16.2±2.8 µA/cm^2^ to 12.7±1.8 µA/cm^2^ (*P*<0.05, n = 4, Figures 2C and 2G). Intriguingly, CQ-evoked increases were almost completely abolished by niflumic acid (10^−4^M), a relatively specific Ca^2+^-activated Cl^−^ channel (CaCC) inhibitor (Figures 2D and 2H). To further confirm the specific target of CQ, we tested the effect of another two well-known CaCC inhibitors (NPPB and DIDS) on CQ-evoked response in *Isc.* The results showed that NPPB and DIDS also completely blocked the CQ-evoked increase in *Isc* (Figures 2E and 2F).

### Colocalization of CaCC-TMEM16A and Bitter Receptor T2R in Small Intestinal Epithelial Cells

**Figure 3:**
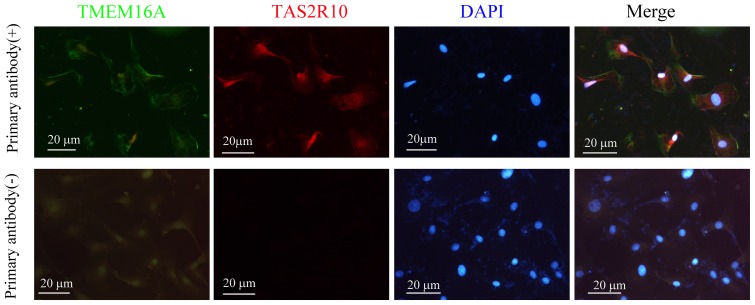
Colocalization of CaCC-TMEM16A and bitter receptor T2R in small intestinal epithelial cells. Immunohistochemistry was showing TMEM16A and bitter receptor TAS2R10 in rat ileum epithelial cell line IEC-18.

To identify whether CaCC or T2R was localized in rat small intestinal epithelial cells, we carried out immunohistochemical study in IEC-18 cells. In our present study, we revealed that T2R and TMEM16A were colocalized in small intestinal epithelial cells (Figure 3).

### Effects of Cl^−^ and Ca^2+^-free Solution on CQ-evoked Increase in *I_SC_* in Rat Ileum

**Figure 4:**
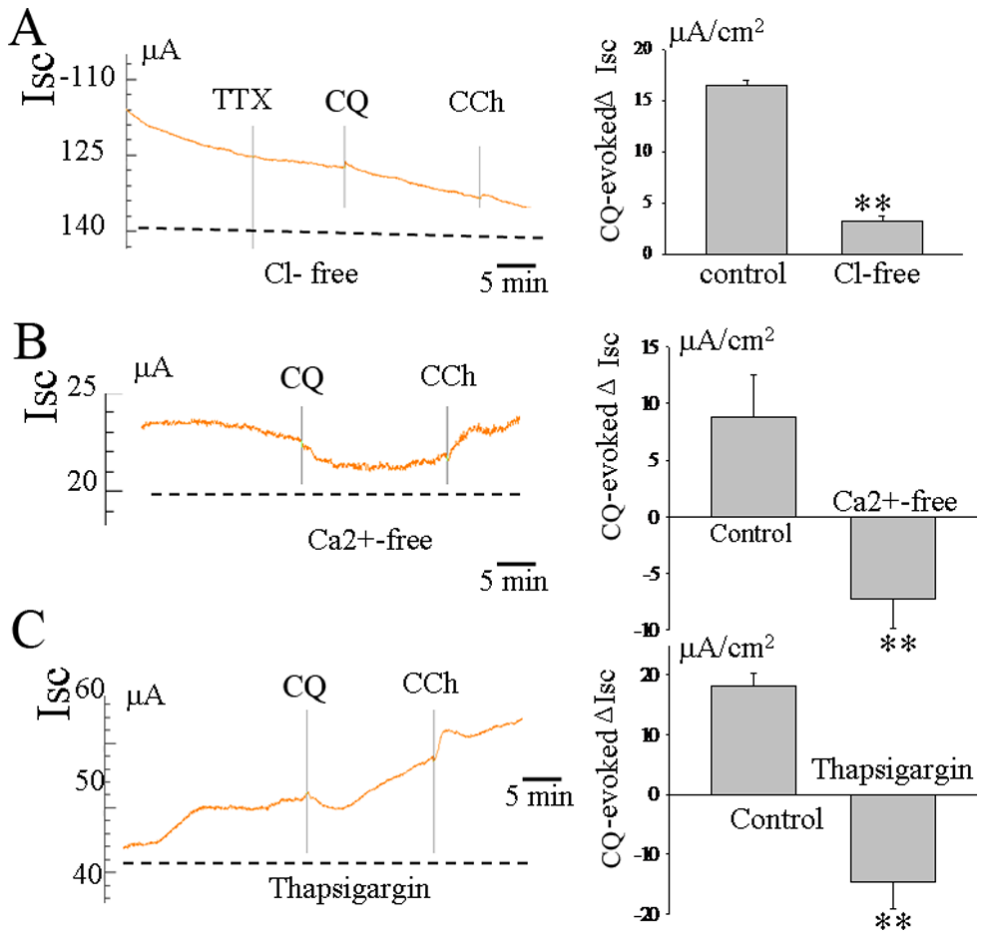
Effects of Cl^−^ and Ca^2+^-free solution on CQ-evoked increase in *I_SC_* in rat ileum. CQ-induced increases in *I_SC_* was greatly reduced in the absence of Cl*^−^*(n = 6,A). In the absence of Ca^2+^, CQ-induced increases in *I_SC_* were totally abolished, whereas a decrease in basal *I_SC_* was seen in this Ca^2+^-free condition(n = 5,B). A similar effect was obtained in response to pretreatment with thapsigargin(10^−6^M), a Ca^2+^ pump inhibitor(n = 6, C). **P<0.01 by unpaired *t*-test.

TAS2R agonists such as saccharin, CQ and denatonium evoke increased intracellular Ca^2+^ concentrations in human airway smooth muscle [6]. In our present study, we also revealed that CQ triggered an increase of intracellular Ca^2+^ concentrations, which would be expected to activate the CaCC and then evoke Cl*^−^* secretion (Figure S1). To further confirm the ionic basis for the increases in *I_SC_* evoked by CQ, Cl^–^free and Ca^2+^-free solutions were used. The serosal CQ-induced *I_SC_* responses were tested in the absence of Cl*^−^* and Ca^2+^ from the Krebs solutions.The CQ-induced increases in *I_SC_* were greatly reduced. (from 16.3±2.2 to 3.1±0.8 µA/cm^2^, *P*<0.01, n = 6, Figure 4A) in the absence of Cl*^−^*. In the absence of Ca^2+^, the CQ-induced increases in *I_SC_* were totally abolished, and a decrease in basal *I_SC_* was seen (n = 5, Figure 4B). Similar results were obtained with thapsigargin(10^−6^M), a Ca^2+^ pump inhibitor(n = 6, Figure 4C).

### Effects of other Bitter Compounds on Basal *I_SC_* in Rat Ileum

**Figure 5:**
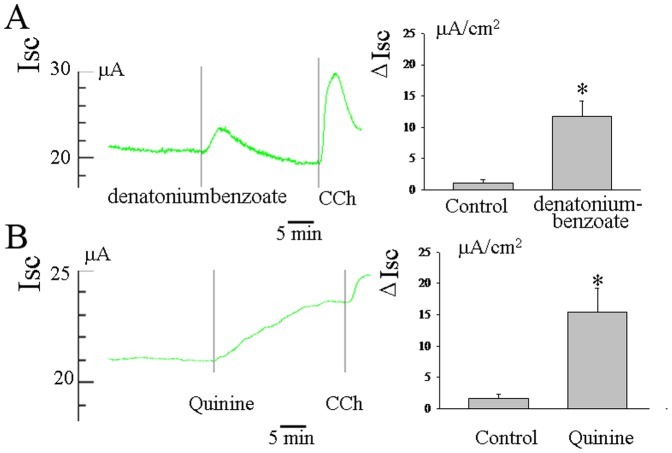
Effect of other bitter compounds such as denatoniumbenzoate and quinine in basal *I_SC_* in rat ileum. Other compounds, such as denatoniumbenzoate and quinine also induced an increase in Isc(A and B). *P<0.05 by unpaired *t*-test.

To examine whether other bitter compounds also evoke secretory responses, we measured the effect of denatoniumbenzoate (10^−4^M) and quinine(10^−4^M) on the basal *I_SC_* in rat ileum as assessed with the Ussing chamber technique. The data showed that the serosal addition of both denatoniumbenzoate and quinine also induced an increase in Isc(n = 5, Figure 5).

## Discussion

In the present study, we demonstrated the action of a bitter taste receptor ligand, CQ, on electrolyte transport in rat ileum. Our results indicate that CQ induces Cl^−^ secretion probably by stimulating CaCC in rat ileum at low concentrations (≤3×10^−4 ^M), and that such effects do not appear to involve a neural pathway.

### CQ Evoked Cl- Secretion in Rat Ileum

CQ(10^−4^M) evoked an increase in basal *I_SC_* in rat ileum. Consistent with previous research [16], addition of CQ (10^−4^M) to the mucosal surface of ileum did not alter basal electrolyte transport, whereas electrolyte transport was affected after addition of CQ to the solution bathing the serosal surface. However, in contrast to our study, these authors reported that addition of CQ (10^−4^M) to the serosal surface reduced *I_SC_* as determined in vitro using a rabbit ileum. Although we also demonstrated that CQ reduced *I_SC_* in rat ileum at high concentration (≥10^−3 ^M), this discrepancy might be attributable to the different animal models used in these two studies. The ileum of different species may have different sensitivity to chloroquine due to the different type of bitter taste receptor or the different expression level of CaCC-TMEM16A. Interestingly, our study revealed that the expression level of CaCC-TMEM16A was different among rabbit and rat(data not shown). Next, we investigated the underlying mechanisms of CQ-evoked increase of *I_SC._* Although our results also supported the concept that Na^+^ absorption was involved in the CQ-induced increase of Isc, the finding that amiloride decreased this response by only 25%, hinted that other(not Na^+^) ion components were involved in the CQ-evoked increased in *Isc*. Since it had been reported that 6-PTU, a similar bitter compound,evokes anion secretion [8], we tested the effects of some Cl^−^ inhibitors,such as CFTR(inh)-172, furosemide, niflumic acid, DIDS and NPPB on CQ-evoked *I_SC_* responses. CFTR is known as an apical membrane Cl^−^ channel in epithelial cells [17], [18]. Our results revealed that CFTR(inh)-172 did not affect the CQ-evoked increase in *I_SC_*_._ The Na^+^-K^+^-2Cl^−^ co-transporter is known as the predominant transporter at basolateral membrane for Cl^−^ uptake into epithelial cells [19]. The intracellular-free chloride concentration is maintained by a Na^+^-K^+^-2Cl^−^ co-transporter that actively accumulates Cl^−^. In the present study, furosemide, a Na^+^-K^+^-2Cl^−^ co-transporter inhibitor, reduced the CQ-evoked *I_SC_* response by 22%. CaCCs are plasma membrane proteins involved in various important physiological processes. In epithelial cells, CaCC activity mediates the secretion of Cl^−^. TMEM16A is recently identified as CaCC [20], [21]. In our present study, we demonstrated that CaCC channel inhibitors, such as niflumic acid, DIDS and NPPB, almost completely abolished the CQ-evoked increase of *I_SC_*, and CaCC-TMEM16A was widely expressed in small intestinal epithelial cells. Given that the CQ-induced increases in *I_SC_* were largerly abolished in Cl^−^-free conditions, it would seem reasonable to conclude that Cl^−^ secretions through CaCC are involved in the *I_SC_* response to CQ.

### The Role of Ca^2+^

**Figure 6:**
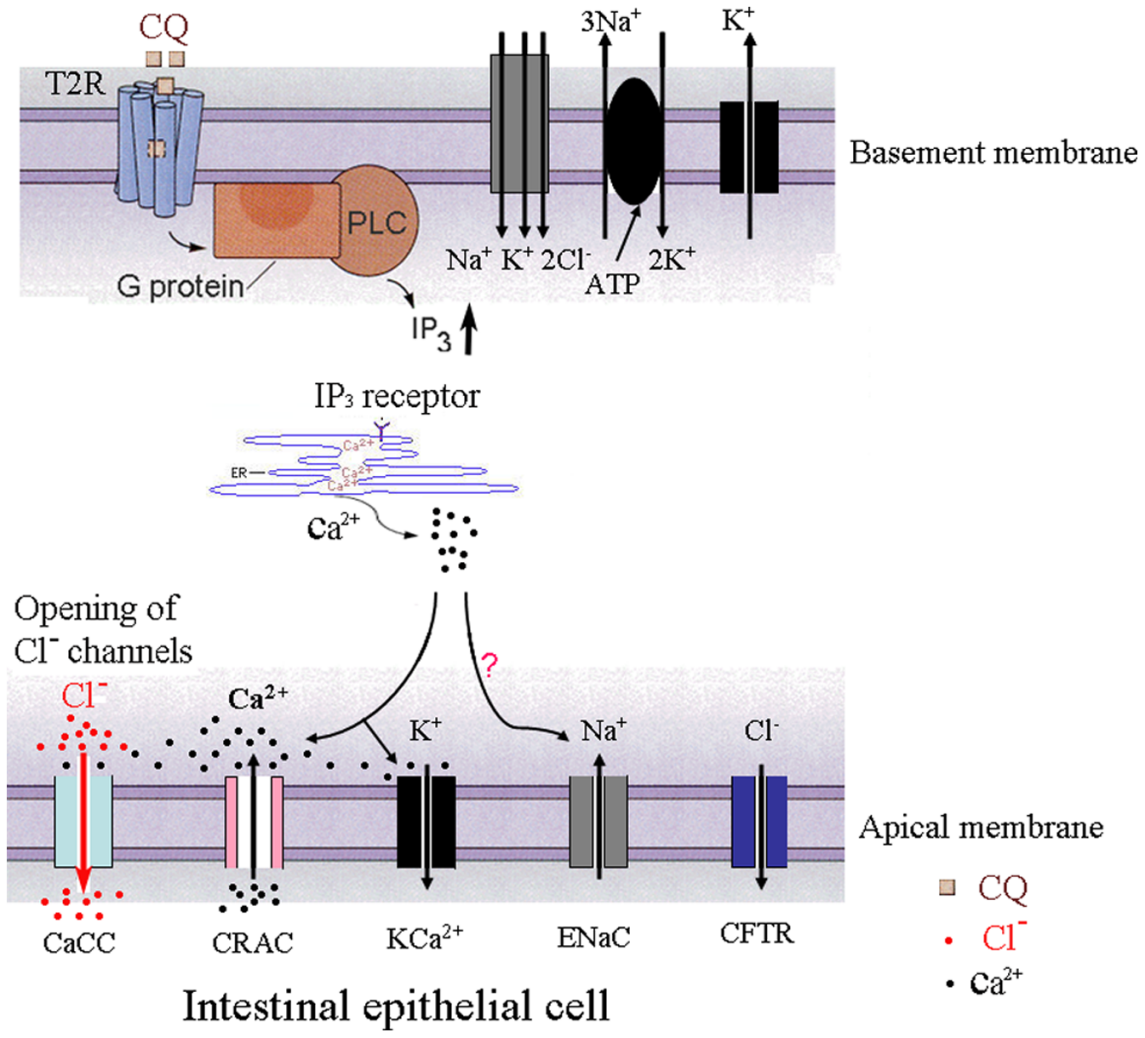
A working model of CQ in intestinal epithelial cells. CQ binds to T2Rs activating a G-protein to produce phospholipase C (PLC) in the basement membrane of intestinal epithelial cells followed by Ca^2+^ release from the sarcoplasmic reticulum (SR). Local Ca^2+^ entry through store-operated Ca^2+^ release-activated Ca^2+^ channels(CRAC) drives Cl*^−^* secretion by stimulating Ca^2+^-activated Cl*^−^* channels(CaCC) in the apical membrane of intestinal epithelial cells. The intracellular-free chloride concentration is maintained by a Na^+^-K^+^-2Cl^−^ co-transporter that actively accumulates Cl^−^.

Based upon our results, it appears that the CQ-evoked increase in *I_SC_* was not dependent on the neural pathway. CQ increases rabbit ileal Ca^2+^ content [16] and evokes increased intracelluar Ca^2+^ of rat small epithelial cells, which suggest that this effect may involve intracelluar Ca^2+^. The bitter taste receptor,T2R, is expressed not only in the taste buds but also in intestinal tissues and we have reported that T2Rs are widely distributed in rat ileum epithelium [13]. In our present study, we revealed that T2R and TMEM16A were colocalized in small intestinal epithelial cells. T2Rs belong to the family of G protein coupled receptors(GPCRs) and activate phospholipase C (PLCβ2), to produce inositol-1,4,5-trisphosphate (IP_3_) and diacylglycerol (DAG) [22]. IP_3_ binds to IP_3_ receptors and elicits a release of Ca^2+^ from the sarcoplasmic reticulum (SR) [23]. Moreover, taste receptor cell responses to the bitter stimulus, denatonium, involve Ca^2+^ influx via store-operated channels [24]. It has been reported that CQ increases rabbit ileal calcium content [16] and increases intracellular Ca^2+^ concentrations [6], [9]. The present results showed that the CQ-evoked *I_SC_* increase in ratileum was highly dependent on either intracellular or extracellular calcium. Therefore, a possible underlying mechanism of this CQ-evoked secretory response is that intestinal epithelial cell responses to this bitter simulus enables Ca^2+^ entry via store-operated Ca^2+^ release-activated Ca^2+^ channels(CRAC) that drives Cl*^−^* secretion by activating CaCC (Figure 6). Other bitter compounds, such as quinine and denatoniumbenzoate had the same effect as CQ. However, it remains unclear which types of T2R are activated by CQ. Interestingly in the absence of extracellar Ca^2+^ or in the presence of thapsigargin, CQ dereased basal *I_SC_*. As mentioned above, high concentration of CQ also induced a decrease in *I_SC_*. These results suggest that the Ca^2+^-activated Cl^−^ channel may not be the only target of CQ. It is known that CQ is a membrane-stabilizer and can be trapped in the cell. Thus the CQ-evoked decrease in *I_SC_* under these conditions may represent a non-specific action of this bitter compound.

In conclusion, the present results indicate that within the rat ileum, Cl*^−^* secretion induced by CQ at low concentrations involved CaCC through a T2R chemical-sensing mechanism in rat ileum. Such effects do not appear to be dependent on a neural pathway. Taken together, our results suggest that fluid secretion stimulated by CQ-induced Cl*^−^* secretion appears to be responsible for gastrointestinal side-effects of this bitter compound in the treatment of malaria.

## Supporting Information

Figure S1 **CQ evoked an increase in intracellular Ca^2+^ in rat ileum epithelial cell line IEC-18 by single cell Ca^2+^ imaging analysis.** *P<0.05; compared with control by paired *t*-test.
(TIF)
