# Supplementary figures and images for: Chloroquine Stimulates Cl− Secretion by Ca2+ Activated Cl− Channels in Rat Ileum
Source: PLoS One. 2014 Jan 30;9(1):e87627. doi: 10.1371/journal.pone.0087627 (PMC3907514; doi:10.1371/journal.pone.0087627)

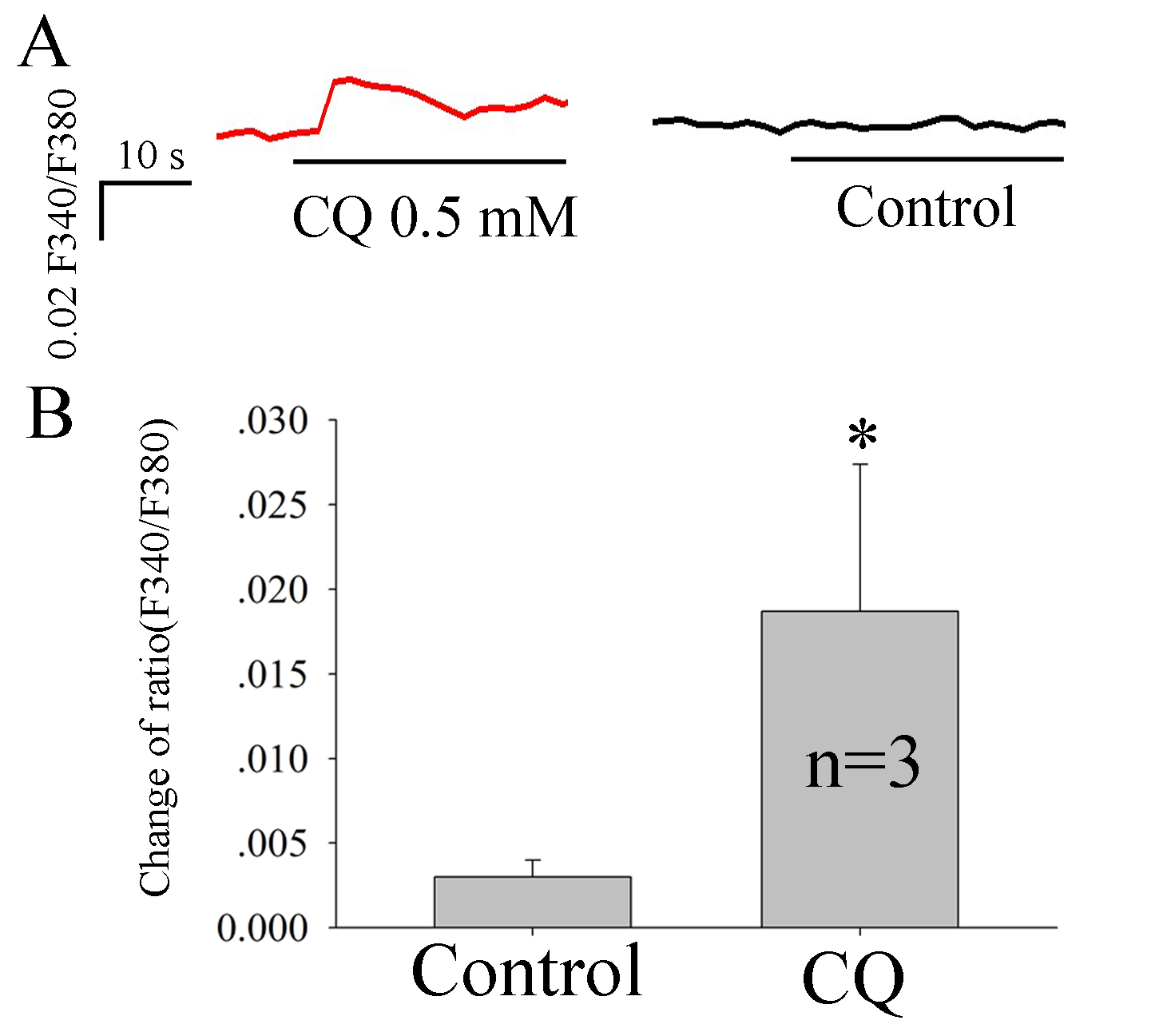

Supplement: Figure S1 — CQ evoked an increase in intracellular Ca2+ in rat ileum epithelial cell line IEC-18 by single cell Ca2+ imaging analysis. *P<0.05; compared with control by paired t-test. (TIF) [file pone.0087627.s001.tif]
